# Acute‐On‐Chronic Liver Failure With Discordant Severity Assessments Following Binge Drinking: A Case Report

**DOI:** 10.1002/jgf2.70165

**Published:** 2026-09-18

**Authors:** Minami Seki, Tomoyuki Watanabe, Yuma Haraguchi

**Affiliations:** ^1^ Division of Internal Medicine Health Co‐op. Watari Hospital Fukushima Japan; ^2^ Division of Cardiology and Internal Medicine Health Co‐op. Watari Hospital Fukushima Japan; ^3^ Division of General Medicine Health Co‐op. Watari Hospital Fukushima Japan

**Keywords:** acute‐on‐chronic liver failure, alcohol use disorder, alcohol‐related cirrhosis, brief intervention, hyperlactatemia

## Abstract

Acute‐on‐chronic liver failure (ACLF) is associated with high short‐term mortality. We report a man in his 50s with alcohol‐related cirrhosis who developed ACLF following binge drinking. Despite being classified as grade 1 ACLF according to the Japanese severity classification, he presented with severe metabolic acidosis and marked hyperlactatemia and died 4 h after admission. Another prognostic scoring system classified him in the most severe category. This case illustrates that different severity assessments may provide complementary information regarding ACLF severity and prognosis and underscores the importance of repeatedly addressing problematic alcohol use and facilitating engagement in specialized addiction treatment during outpatient care.

## 1 Background

Acute‐on‐chronic liver failure (ACLF) is characterized by acute deterioration of chronic liver disease, resulting in multiorgan failure and high short‐term mortality [1].

Precipitating factors include infection, gastrointestinal bleeding, and excessive alcohol consumption, particularly in alcohol‐related liver disease [2]. Although ACLF severity is generally assessed according to organ failure, additional prognostic factors may provide complementary information. Continued alcohol use remains an important preventable precipitating factor.

We report a case of ACLF following binge drinking in a patient with alcohol‐related cirrhosis who died rapidly despite being classified as grade 1 ACLF according to the Japanese severity classification. This case illustrates the complementary value of different severity assessments and suggests the importance of repeatedly addressing problematic alcohol use during outpatient care.

## 2 Case Report

A man in his 50s presented to the emergency department with dyspnea and vomiting. He had been intermittently followed at our outpatient clinic for liver dysfunction and subsequently alcohol‐related cirrhosis since 2016. His Child–Pugh score was 9. His alcohol consumption had been poorly controlled, and he frequently interrupted follow‐up. Progression of cirrhosis and ascites had been noted, and he had a history of endoscopic variceal ligation (EVL) for esophageal variceal rupture.

On June 14, 2025, he engaged in heavy alcohol consumption at a barbecue and continued drinking thereafter with minimal oral intake. Late at night on June 18, he developed nausea and vomiting followed by worsening dyspnea and was transported to the hospital by ambulance.

On arrival, his consciousness was fluctuating, with a Glasgow Coma Scale score of E3V3M6. His vital signs were blood pressure 130/76 mmHg, heart rate 152 beats/min, body temperature 35.6°C, and respiratory rate 30 breaths/min. He had marked scleral jaundice, abdominal distension, peripheral coldness, and mild asterixis.

Laboratory tests revealed marked hyperbilirubinemia, renal dysfunction, elevated inflammatory markers, hyperammonemia, hypoglycemia, and coagulopathy. Marked hyperlactatemia and severe metabolic acidosis were also present (Table 1). Urinalysis showed bilirubinuria and pyuria. Contrast‐enhanced computed tomography showed no pulmonary embolism or superior mesenteric artery occlusion. Only intestinal wall thickening and ascites were observed.

**TABLE 1:** Laboratory findings on admission.

WBC	119 × 10^2^	/μL	NH_3_	289	N‐μg/dL	PT‐INR	1.77	
RBC	450 × 10^4^	/μL	Na	140	mEq/L	APTT	44.7	Sec
Hb	14.1	g/dL	K	4.2	mEq/L	FDP	113.2	μg/mL
Hct	36.3	%	Cl	95	mEq/L	PCT	0.36	ng/mL
Plt	21.2 × 10^4^	/μL	Ca	9.0	mg/dL	Arterial blood gas analysis		
ALP	165	IU/L	iP	8.7	mg/dL	pH	6.937	
AST	309	IU/L	BUN	20.5	mg/dL	PaCO_2_	10.2	mmHg
ALT	66	IU/L	Cr	1.99	mg/dL	PaO_2_	132.0	mmHg
LDH	462	IU/L	eGFR	29.7		HCO^3−^	2.1	mmol/L
γ‐GTP	117	IU/L	UA	8.8	mg/dL	BE	−29.7	mmol/L
CK	300	IU/L	Glucose	56	mgdL	SaO_2_	96.2	%
T‐bil	16.4	mg/dL	HbA1c	3.7	%	Lact	29.0	mmol/L
D‐bil	13.1	mg/dL	CRP	3.57	mg/dL			

Abbreviations: ALP, alkaline phosphatase; ALT, alanine aminotransferase; APTT, activated partial thromboplastin time; AST, aspartate aminotransferase; BE, base excess; BUN, blood urea nitrogen; Ca, calcium; CK, creatine kinase; Cl, chloride; Cr, creatinine; CRP, C‐reactive protein; D‐bil, direct bilirubin; eGFR, estimated glomerular filtration rate; FDP, fibrin/fibrinogen degradation products; glucose, plasma glucose; Hb, hemoglobin; HbA1c, hemoglobin A1c; HCO₃⁻, bicarbonate; Hct, hematocrit; K, potassium; Lact, lactate; LDH, lactate dehydrogenase; Na, sodium; NH₃, ammonia; PaCO₂, partial pressure of carbon dioxide; PaO₂, partial pressure of oxygen; PCT, procalcitonin; pH, potential of hydrogen; Plt, platelet count; PT‐INR, prothrombin time–international normalized ratio; RBC, red blood cell count; SaO₂, arterial oxygen saturation; T‐bil, total bilirubin; UA, uric acid; WBC, white blood cell count; γ‐GTP, gamma‐glutamyl transpeptidase.

According to the Japanese diagnostic criteria for ACLF [3], the patient fulfilled the criteria based on underlying cirrhosis, recent excessive alcohol consumption, a total bilirubin level of 16.4 mg/dL, and a PT‐INR of 1.77. He was classified as grade 1 ACLF because only the criterion for liver failure was met (total bilirubin ≥ 12 mg/dL). Although renal dysfunction was present, the creatinine level of 1.99 mg/dL did not meet the threshold for renal failure (≥ 2.0 mg/dL). Central nervous system failure could not be definitively established because his altered consciousness was potentially multifactorial.

For calculation of the AARC score, his altered consciousness was considered equivalent to grade II hepatic encephalopathy (2 points). Together with the corresponding scores for total bilirubin (2 points), PT‐INR (1 point), serum lactate (3 points), and serum creatinine (3 points), the total AARC score was 11, placing him in the most severe category (AARC grade III) [4].

During outpatient follow‐up, the patient had repeatedly declined referral for advanced medical care despite progressive clinical deterioration, and his wishes had been documented in the medical record. At his final admission, based on his previously expressed wishes and those of his family, transfer to a tertiary care center and escalation of treatment were not pursued. Consequently, blood cultures, vitamin measurements, and antimicrobial therapy were not performed. Despite fluid resuscitation and sodium bicarbonate, he died approximately 4 h after admission.

## 3 Discussion

ACLF is characterized by acute deterioration of chronic liver disease, leading to multiorgan failure and high short‐term mortality, with mortality increasing with the severity and number of organ failures [5]. The pathophysiology of ACLF involves alterations in the gut microbiota, increased intestinal permeability, and systemic inflammation triggered by acute precipitating factors, ultimately leading to multiorgan failure [6].

Although the patient was classified as grade 1 ACLF according to the Japanese severity classification [3], his condition was severe at presentation. The AARC score, which incorporates total bilirubin, hepatic encephalopathy, PT‐INR, serum lactate, and creatinine to predict 28‐day mortality, was 11 points, corresponding to the most severe category [4]. In this patient, the AARC score provided additional prognostic information by incorporating clinical and laboratory variables, including the markedly elevated serum lactate level. These findings suggest that assessment using different scoring systems may provide complementary information for evaluating the severity and prognosis of ACLF.

Elevated lactate can result from tissue hypoperfusion or hypoxia, impaired clearance associated with hepatic or renal dysfunction, and medications such as metformin [7]. In this case, marked hyperlactatemia was likely multifactorial, reflecting impaired clearance due to liver failure, hypoperfusion associated with dehydration, metabolic disturbances related to heavy alcohol consumption and malnutrition, and possible concomitant infection.

Tachypnea and altered mental status corresponded to a qSOFA score of 2, and marked hyperlactatemia was also present, raising the possibility of concomitant sepsis. However, qSOFA is not diagnostic of sepsis, and hyperlactatemia is nonspecific. Furthermore, there was no definitive evidence of infection, and blood cultures were not obtained or antimicrobial therapy administered. Therefore, sepsis could neither be confirmed nor excluded, and its contribution to the rapid deterioration remains uncertain.

**FIGURE 1:**
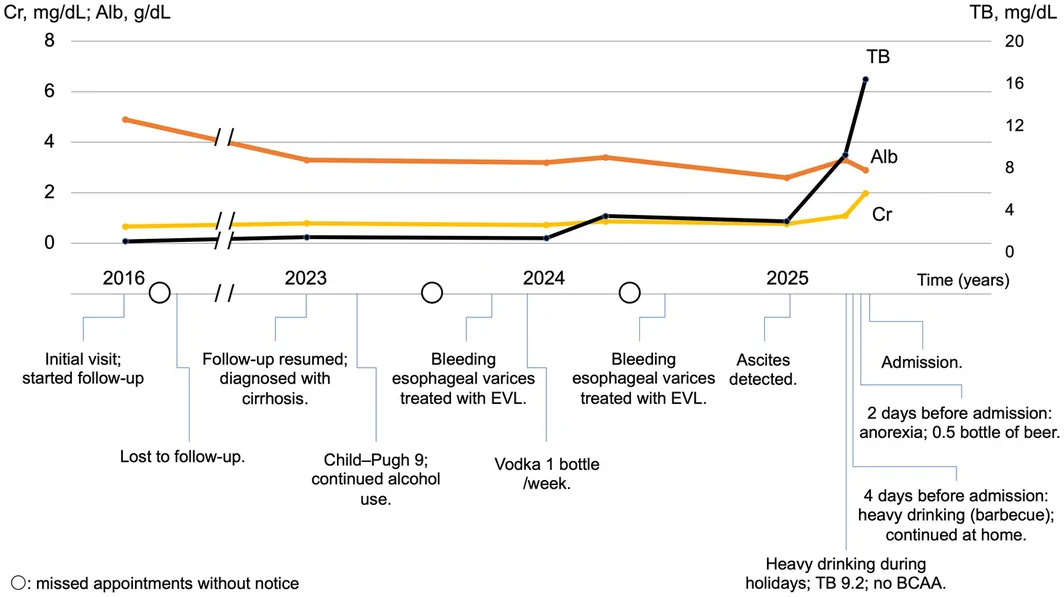
Clinical course from 2016 to hospitalization in 2025. The upper section depicts longitudinal changes in total bilirubin, creatinine, and albumin levels. Total bilirubin began to increase from 2024, while albumin showed a gradual declining trend over time; both parameters deteriorated rapidly immediately prior to hospitalization in 2025. The lower section outlines key clinical events during outpatient follow‐up. Despite repeated missed visits, the patient continued outpatient follow‐up for liver dysfunction. Several days before hospitalization, excessive alcohol intake occurred, which was considered the precipitating factor leading to admission. Open circles indicate missed appointments without prior notice. A break in the x‐axis (/ /) indicates the omitted interval between 2016 and 2023.

From a preventive perspective, this case raises the question of whether earlier intervention for continued alcohol use might have reduced the risk of ACLF. During 9 years of intermittent outpatient follow‐up, there had been repeated opportunities to address his problematic alcohol use before ACLF developed (Figure 1).

Brief interventions by physicians have been shown to reduce alcohol consumption and alcohol‐related behaviors in outpatient settings [8]. In this patient, repeated explanations regarding the risks of continued drinking did not result in sustained behavioral change. More structured approaches, such as motivational interviewing, might have supported readiness for change, although their effects are generally modest and may be insufficient alone in patients with established alcohol dependence [9].

Another challenge was the gap between continued general medical care and engagement in specialized addiction treatment. Although the patient intermittently attended our clinic, he repeatedly declined referral to psychiatric or addiction services. Sustained engagement in addiction treatment remains difficult even when screening, brief intervention, and treatment initiation are implemented in primary care [10]. Although it is uncertain whether additional intervention would have prevented ACLF in this patient, this case underscores the need to continue addressing alcohol use nonjudgmentally during routine care and to offer opportunities for engagement with specialized addiction treatment.

## 4 Conclusion

This case illustrates that different severity assessments may provide complementary prognostic information in ACLF and suggests a potential role for continued efforts to address problematic alcohol use during outpatient care.

## Author Contributions

**Yuma Haraguchi:** supervision. **Minami Seki:** writing – original draft. **Tomoyuki Watanabe:** writing – review and editing.

## Funding

The authors have nothing to report.

## Ethics Statement

This study was conducted in accordance with the principles of the Declaration of Helsinki.

The study was approved by the institutional review board (approval number: 2026−001).

## Consent

Written informed consent was obtained from the patient's family.

## Conflicts of Interest

The authors declare no conflicts of interest.

## Data Availability

The data that support the findings of this study are not publicly available due to patient privacy concerns but are available from the corresponding author upon reasonable request.
